# An Extremely Rare and Aggressive Case of Undifferentiated Carcinoma With Osteoclast-Like Giant Cells of the Pancreas Presenting as a Paraneoplastic Syndrome: A Case Report and Literature Review

**DOI:** 10.7759/cureus.51996

**Published:** 2024-01-10

**Authors:** Mohammed N AlAli, Ghada I Alothman, Mohamed S Essa, Muath Alrashed, Sadiq M Amer, Farah ALMuqrin, Abdullah M Albdah, Ossama Alamri

**Affiliations:** 1 Department of Surgery, Prince Mohammed Bin Abdulaziz Hospital, Ministry of Health, Riyadh, SAU; 2 College of Medicine, King Saud University, Riyadh, SAU; 3 Department of Pathology, Prince Mohammed Bin Abdulaziz Hospital, Ministry of Health, Riyadh, SAU

**Keywords:** malignancy, dermatomyositis, paraneoplastic syndrome, pancreatic cancer, undifferentiated carcinoma with osteoclast-like giant cells

## Abstract

Undifferentiated carcinoma with osteoclast-like giant cells (UC-OGC) is a rare tumor type of pancreatic cancer. Paraneoplastic syndromes, an idiopathic inflammatory myositis characterized by various skin manifestations (such as dermatomyositis (DM)), cannot be attributed to the primary tumor itself. Here, we report an unusual case of UC-OGC presenting as a paraneoplastic syndrome, the first reported from Saudi Arabia and the Arabian Gulf states. A 49-year-old Eritrean woman with known DM was referred to our hospital with a left-sided pleural effusion. Computed tomography of the abdomen revealed a large necrotic splenic mass (~17 × 12.9 × 18.2 cm). The patient underwent exploratory laparotomy with en bloc resection of the mass (splenectomy, distal pancreatectomy, and partial excision of the left hemidiaphragm). Following a histopathological examination of the mass, UG-OGC of the pancreas, presenting as a paraneoplastic syndrome, was diagnosed. To our knowledge, this case is the first to present a paraneoplastic syndrome associated with UC-OGC. The identification of an exceedingly rare tumor presenting atypically as a paraneoplastic syndrome shows the importance of conducting comprehensive examinations of patients with malignancies, emphasizing the need for more reports of similar cases.

## Introduction

Undifferentiated carcinoma with osteoclast-like giant cells (UC-OGC) is a rare tumor accounting for fewer than 1% of all pancreatic cancers [1]. According to the World Health Organization classification, UC-OGC is a clinically and morphologically distinct subtype of pancreatic ductal adenocarcinoma [2]. UC-OGC commonly presents in women aged >50 years, with atypical symptoms such as upper abdominal pain, weight loss, anorexia, jaundice, and steatorrhea [3]. It usually occurs in the body and tail of the pancreas, and the gold standard for diagnosis includes immunohistochemical and histopathological studies [3]. Notably, lymph involvement and distant metastases are rarely observed in UC-OGC [3], and en bloc resection is considered the first line of treatment. However, the prognosis for these patients remains controversial [4,5]. Paraneoplastic syndromes are conditions that cannot be attributed to the primary tumor itself, its metastases, or hormones produced by the affected tissues [6,7]. These are mainly categorized into four groups according to the symptoms, namely, endocrinological, hematological, neurological, or dermatological symptoms [6,7]. Dermatomyositis (DM) is a rare idiopathic inflammatory myositis characterized by various skin manifestations [8]. It is more common in women than in men [9] and can be associated with malignancies such as ovarian, lung, breast, and head and neck cancer, as well as non-Hodgkin lymphoma [10].

In this study, we report an interesting, rare, and unusual case of UC-OGC presenting as a paraneoplastic syndrome. To our knowledge, this case is the first of its type reported from Saudi Arabia and the Arabian Gulf states.

## Case presentation

A 49-year-old Eritrean woman with four-year and one-year known diagnoses of Graves’ disease and DM, respectively, was referred to our hospital for further assessment and management after receiving a diagnosis of left-sided pleural effusion using chest radiography. The patient had started experiencing left upper abdominal pain approximately two months before the presentation. The pain was referred to the left shoulder and was of insidious onset, being intermittent, progressive, and twisting in nature. It was associated with abdominal distention, fever of no specific pattern, anorexia, nausea, generalized fatigue, night sweats, unintentional weight loss (approximately 13 kg in two months), and a skin rash over her cheeks and the front and back of her neck. Notably, the patient had no family history of rheumatological or malignant diseases.

Upon examination, the patient appeared cachectic and ill; however, she was vitally stable on room air and had an oral temperature of 36.8°C, peripheral pulse rate of 97 beats/minute, blood pressure of 114/75 mmHg, and SpO_2_ of 95%. The patient had hyperpigmented skin rashes over her cheeks and the front and back of her neck (V and Shawl signs, respectively). Furthermore, skin hypopigmentation (Gottron’s papules) was observed on both knuckles. Upon palpation, the abdomen was slightly distended and tender in the left hypochondrial, epigastric, and left lumbar regions. Moreover, the liver was palpable 4 cm below the costal margin on the middle to mid-axillary line, with a firm consistency, and the spleen was enlarged and solid. Notably, the chest showed decreased breath sounds in the left area. Initial laboratory analyses showed leukocytes of 16.3 × 10^9^/L (reference range: 4.0-11.0 × 10^9^/L), hemoglobin of 96.0 g/L (reference range: 120.0-115.0 g/L), platelets of 510 × 10^9^/L (reference range: 150-450 × 10^9^/L), albumin of 34 g/L (reference range: 35-52 g/L), urea of 1.3 mmol/L (reference range: 2.5-6.7 mmol/L), creatinine of 47.40 μmol/L (reference range: 53.00-97.00 μmol/L), C-reactive protein of 9.21 mg/dL (reference range: 0.01-0.50 mg/dL), creatine kinase of 11 U/L (reference range: 29-168 U/L), lactate dehydrogenase of 951 U/L (reference range: 125-243 U/L), anti-double stranded DNA of 83.25 IU/mL (reference range: 0-75 IU/mL), anti-SSA of 3.48 U (reference range: 0-0.9 U), and anti-SSB of 1.7 U (reference range: 0-0.9 U), as well as the international normalized ratio of 1.19 (reference range: 0-1.1) and erythrocyte sedimentation rate of 79 mm/hour (reference range: 1-20 mm/hour). Due to the patient’s history of bilateral changes in the eyelids, proximal muscle weakness, inability to comb her hair, inability to stand from a bed or chair, and an 18-month history of mouth and eye dryness, myasthenia gravis was suspected, and the rheumatology service was consulted. Thereafter, the patient was diagnosed with DM, most likely paraneoplastic, based on muscle weakness, a heliotrope rash, Gottron’s papules, and a V-shaped rash. Therefore, the patient was initiated on high-dose steroids and mycophenolate mofetil, which resulted in improvement in all symptoms except for the skin rash.

**Figure 1:**
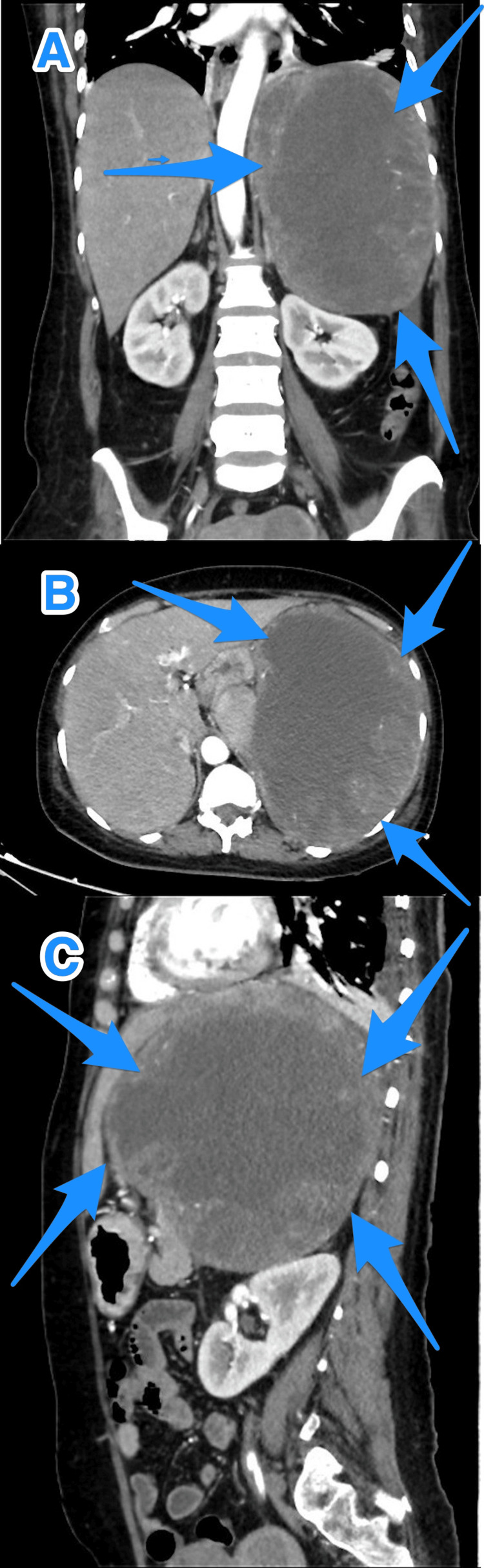
A multiview computed tomography scan of the abdomen (A-C) showing a large mass occupying the left upper quadrant of the abdomen, with no clear origin or borders between the pancreas, spleen, and stomach (arrows).

The patient then underwent ultrasonography of the abdomen, which revealed a large solid mass (approximately 15 × 14 cm) with a cystic area in the left hypochondrium, which was related to the left lobe of the liver and spleen. Further investigation using computed tomography (CT) of the abdomen showed a large necrotic splenic mass with multiple peripheral vascularities of the soft tissue component (approximately 17 × 12.9 × 18.2 cm), as well as possible invasion of the adjacent structures (Figure 1). Further CT of the chest showed no signs of metastasis, and pleural fluid tapping showed only inflammatory cells and no malignancy. Moreover, an ultrasound-guided core biopsy of the left upper abdominal mass revealed poorly differentiated malignant neoplasm.

**Figure 2:**
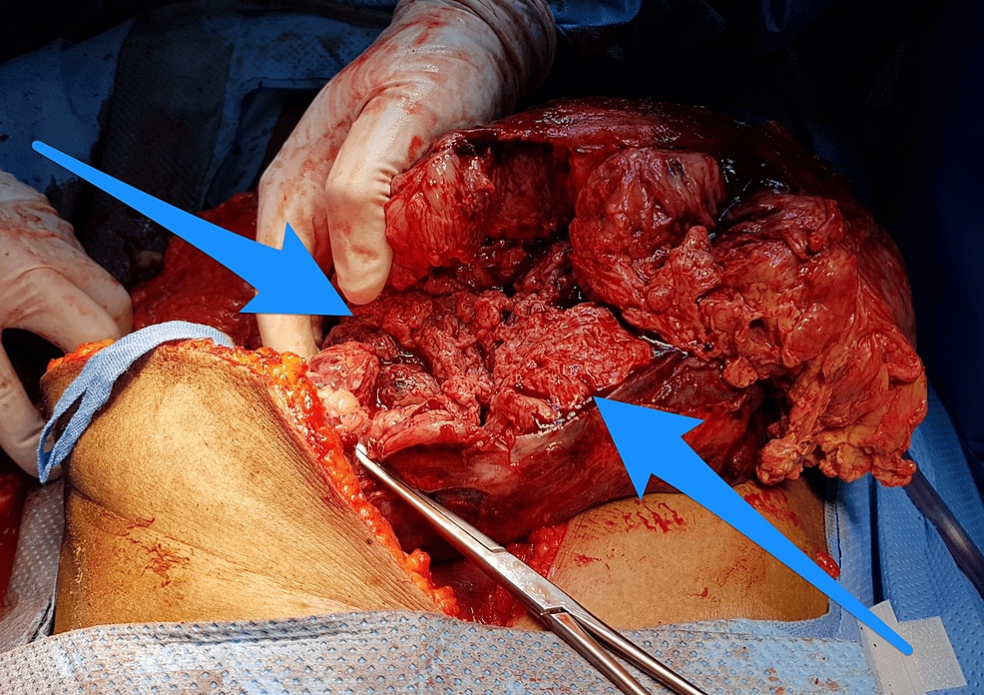
Intraoperative finding of a large mass at the tail of the pancreas and spleen (arrows).

**Figure 3:**
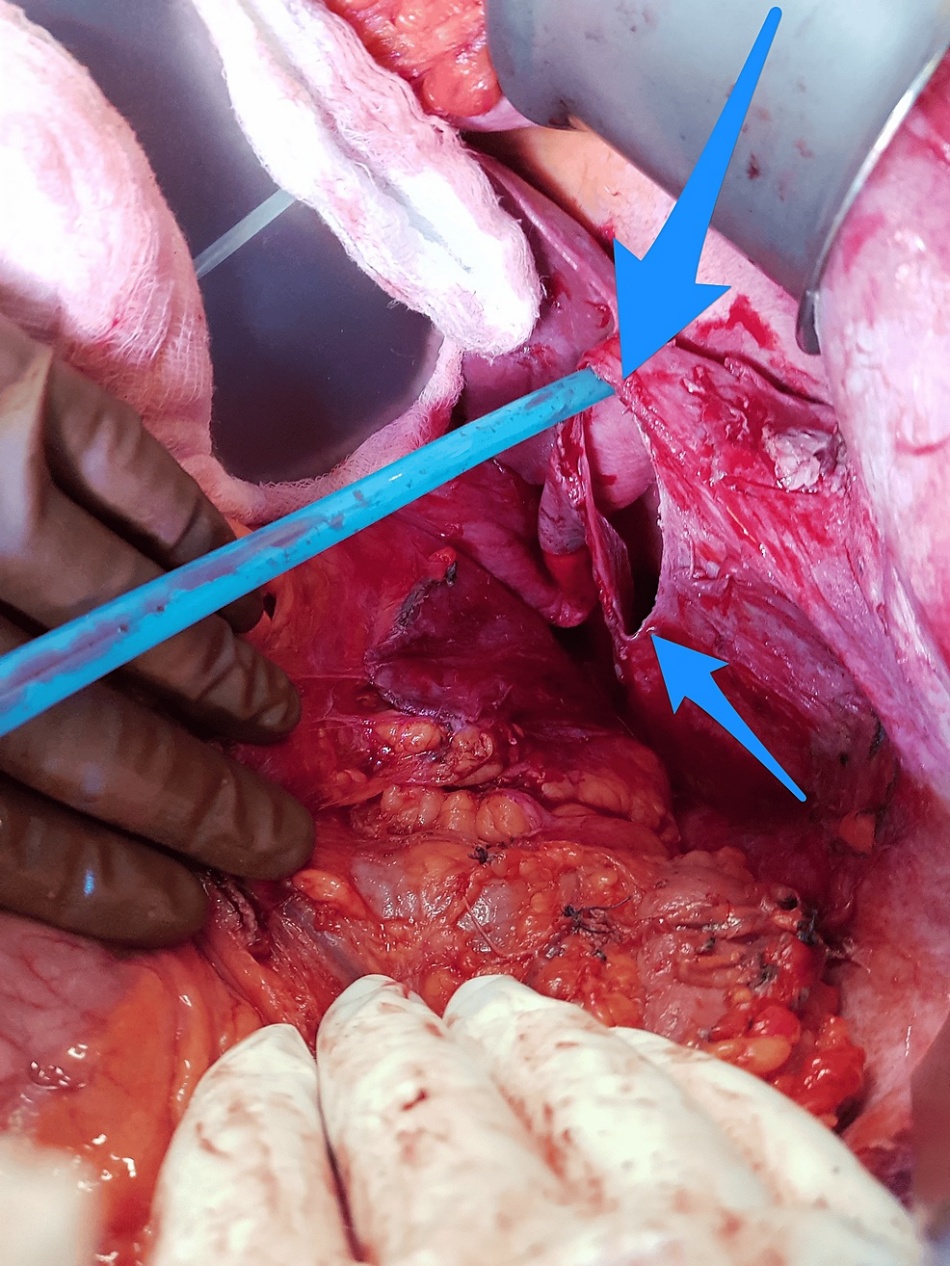
Intraoperative view of the partial diaphragmatic resection (arrows).

**Figure 4:**
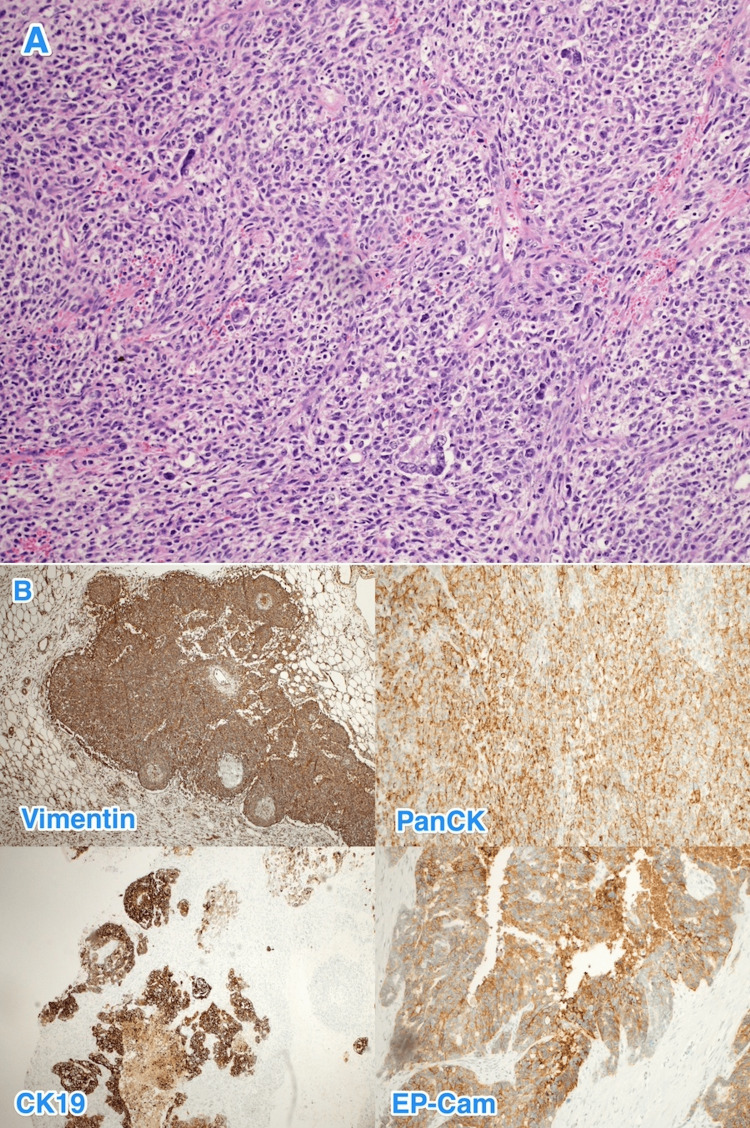
Histopathology of pancreatic neoplasm. The histological sections show (A) a tumor composed of three cell types, namely, mononuclear neoplastic cells, mononuclear histiocytes, and multinucleated giant cells (osteoclast-like giant cells), in a background of hemorrhage and necrosis. The neoplastic cells (B) are positive for vimentin, PanCK, CK19, and EP-Cam while negative for CD45, synaptophysin, and chromogranin (hematoxylin and eosin stain; 10×).

The core biopsy results led to a diagnosis of a poorly differentiated malignant neoplasm. However, serum tumor markers were not available. Therefore, the case was discussed with a multidisciplinary tumor board and surgical intervention was planned. The patient underwent an exploratory laparotomy with en bloc resection of the mass (splenectomy, distal pancreatectomy, and partial excision of the left hemidiaphragm) and left chest tube insertion. The intraoperative finding was a large left hypochondrial mass extending retropancreatically and adhering to the tail of the pancreas (Figures 2, 3). Histopathological examination of the resected specimen revealed an undifferentiated (anaplastic) carcinoma of the pancreas with osteoclast-like giant cells (Figure 4). The mass was located in the tail of the pancreas (approximately 15 × 14 × 10 cm) and had a pathological stage of T3N0Mx; therefore, UC-OGC of the pancreas presenting as a paraneoplastic syndrome was diagnosed.

Postoperatively, a suspicious increase in drain output occurred, suggesting a biochemical leak; therefore, the patient was managed conservatively. After improvement in the drain output, a follow-up CT scan of the abdomen revealed no collection. Overall, the patient experienced no other postoperative complications. Following a postoperative discussion with the multidisciplinary tumor board, the patient was referred to another medical center for adjuvant chemotherapy and further care. However, the patient left the country with no clear follow-up information regarding recurrence and complications.

## Discussion

UC-OGC is a rare subtype of pancreatic adenocarcinoma. In our literature review, we found no reported cases of UC-OGC in Saudi Arabia or the Arabian Gulf states; therefore, this case was rare and novel. Generally, the mean age of patients presenting with UC-OGC is 62 years, with a broad age range of 32-93 years [2]. Additionally, patients with UG-OGC are predominantly female, with a male-to-female ratio of 8:13 [3]. However, data regarding the clinicopathological features and prognosis of UG-OGC are scarce. The survival duration of patients with UC-OGC ranges from a few months to 14 years [11] and is significantly better than that of pancreatic ductal adenocarcinomas without osteoclastic cells [12]. Unfortunately, the patient in the present case was lost to follow-up and we could not determine survival.

The most common presentation of UC-OGC located in the head of the pancreas is jaundice and weight loss, whereas patients with tumors located in the body and/or tail of the pancreas more commonly present with abdominal pain, as observed in our patient [13]. In addition, the levels of tumor markers such as carcinoembryonic antigen and cancer antigen 125 are usually normal or slightly elevated [14]. In the present case, serum tumor markers were not available at our center at the time of diagnosis. Therefore, we used CT or endoscopic ultrasound-guided fine-needle aspiration (FNA), which helps in the diagnosis of tumor cytology. However, preoperative FNA may increase the incidence of postoperative complications and lower the long-term survival rate of patients [15]. Macroscopically, UC-OGCs are characterized by their large size, reaching 5-10 cm at the time of diagnosis [12,15]. This is consistent with our case, which revealed a large tumor measuring approximately 17 × 12.9 × 18.2 cm. Most UC-OGCs are located in the pancreatic body or tail [3]; similarly, the tumor adhered to the pancreatic tail in our case. Moreover, conventional UC-OGC cell types include non-neoplastic osteoclast-like multinucleated giant cells, mononuclear histiocytic components, and neoplastic mononuclear cell components [2].

Owing to its rarity, no standardized treatment for UC-OGC exists and the efficacies of chemotherapy and/or radiation therapy have not been well studied. However, some studies have suggested the use of a similar surgery to that for ductal pancreatic carcinoma as the standard treatment because it is a variant of UC-OGC. The first line of treatment for ductal pancreatic carcinoma is surgical resection with or without concomitant chemotherapy and/or radiation therapy [14]. In this case, we opted for en bloc resection, including splenectomy, distal pancreatectomy, and partial excision of the left hemidiaphragm. However, several studies have reported poor postoperative outcomes using this method, and there is limited data regarding the role of adjuvant chemotherapy and radiotherapy given the rarity of this tumor [4]. In most cases, there is early recurrence and rapid progression despite complete surgical resection, and most patients die within one year postoperatively [3]. In our case, postoperative assessment confirmed that the staging was consistent with the literature (T3N0M0), as lymph involvement and distant metastasis are rarely observed in UC-OGC [3].

Our patient also had DM, which has characteristic skin manifestations such as heliotrope rash, Gottron’s papules, cuticular changes (periungual telangiectasia, photo-distributed erythema, or poikiloderma), and scaly alopecia [8]. Screening for malignancy is crucial in cases with DM, as there is a significant risk of malignancy, even among younger patients, in patients with DM compared to the general population [9], as in the current case. The standard treatment for DM includes strict photoprotection, topical corticosteroids, and immunosuppressive agents [16]. Similar to paraneoplastic syndromes, it often regresses with surgical treatment of the underlying neoplasm and reappears with recurrence [17]. To our knowledge, no cases of paraneoplastic syndromes associated with UC-OGC have been reported; therefore, our case is novel.

## Conclusions

Identifying an exceedingly rare tumor such as an UC-OGC of the pancreas presenting as a paraneoplastic syndrome in an atypical manner underscores the importance of conducting comprehensive examinations of patients with malignancies. Notably, unlike previously reported cases, the tumor reported in our case was very large, and we acknowledge the need for the dissemination of more reports of similar cases.

## References

[1] Pan ZG, Wang B (2007). Anaplastic carcinoma of the pancreas associated with a mucinous cystic adenocarcinoma. A case report and review of the literature. JOP.

[2] Nagtegaal ID, Odze RD, Klimstra D (2020). The 2019 WHO classification of tumours of the digestive system. Histopathology.

[3] Cavalcanti E, Schena N, Serino G, Lantone G, Armentano R (2021). Assessment and management of undifferentiated carcinoma with osteoclastic like giant cells of the pancreas: a case report and revision of literature. BMC Gastroenterol.

[4] Abid H, Gnanajothy R (2019). Osteoclast giant cell tumor of pancreas: a case report and literature review. Cureus.

[5] Speisky D, Villarroel M, Vigovich F (2020). Undifferentiated carcinoma with osteoclast-like giant cells of the pancreas diagnosed by endoscopic ultrasound guided biopsy. Ecancermedicalscience.

[6] Ungprasert P, Bethina NK, Jones CH (2013). Malignancy and idiopathic inflammatory myopathies. N Am J Med Sci.

[7] Hudson CN, Curling M, Potsides P, Lowe DG (1993). Paraneoplastic syndromes in patients with ovarian neoplasia. J R Soc Med.

[8] Callen JP (2000). Dermatomyositis. Lancet.

[9] Qiang JK, Kim WB, Baibergenova A, Alhusayen R (2017). Risk of malignancy in dermatomyositis and polymyositis. J Cutan Med Surg.

[10] Fardet L, Dupuy A, Gain M (2009). Factors associated with underlying malignancy in a retrospective cohort of 121 patients with dermatomyositis. Medicine (Baltimore).

[11] Molberg KH, Heffess C, Delgado R, Albores-Saavedra J (1998). Undifferentiated carcinoma with osteoclast-like giant cells of the pancreas and periampullary region. Cancer.

[12] Muraki T, Reid MD, Basturk O (2016). Undifferentiated carcinoma with osteoclastic giant cells of the pancreas: clinicopathological analysis of 38 cases highlights a more protracted clinical course than currently appreciated. Am J Surg Pathol.

[13] Togawa Y, Tonouchi A, Chiku T (2010). A case report of undifferentiated carcinoma with osteoclast-like giant cells of the pancreas and literature review. Clin J Gastroenterol.

[14] Demetter P, Maréchal R, Puleo F (2021). Undifferentiated pancreatic carcinoma with osteoclast-like giant cells: what do we know so far?. Front Oncol.

[15] Reid MD, Muraki T, HooKim K (2017). Cytologic features and clinical implications of undifferentiated carcinoma with osteoclastic giant cells of the pancreas: an analysis of 15 cases. Cancer Cytopathol.

[16] Femia AN, Vleugels RA, Callen JP (2013). Cutaneous dermatomyositis: an updated review of treatment options and internal associations. Am J Clin Dermatol.

[17] Ono K, Shimomura M, Toyota K (2017). Successful resection of liver metastasis detected by exacerbation of skin symptom in a patient with dermatomyositis accompanied by rectal cancer: a case report and literature review. Surg Case Rep.

